# Assessment of myofascial medialization following intraoperative fascial traction (IFT) in a cadaveric model

**DOI:** 10.1007/s10029-024-03003-1

**Published:** 2024-04-14

**Authors:** H. Niebuhr, W. Reinpold, F. Morgenroth, C. Berger, H. Dag, U. Wehrenberg, J. Trzewik, F. Köckerling

**Affiliations:** 1 Hamburg Hernia Centre, Hamburg, Germany; 2 Anatomical Institute University of Hamburg, Hamburg, Germany; 3 Hamm-Lippstadt University of Applied Sciences, Hamm, Germany; 4 Hernia Centre Vivantes Humboldt-Klinikum Berlin, Berlin, Germany

**Keywords:** Ventral hernia repair, IFT, Fascial traction, Cadaveric model, Myofascial advancement, Fasciotens

## Abstract

**Purpose:**

Intraoperative fascial traction (IFT) for the treatment of large ventral hernias and loss of domain (LOD) hernias is a promising tool in abdominal wall surgery. However, little is known about the extent of gain in myofascial advancement especially for the anterior rectus sheath. We, therefore, used a cadaveric model to determine the medialization during IFT.

**Methods:**

4 fresh frozen specimens were used. Retromuscular preparation was carried out followed by IFT with diagonal vertical traction for 30 min. Medial advancement of the anterior rectus sheath was measured after 15 and 30 min as well as traction forces.

**Results:**

Total medialization for anterior rectus sheath after 30 min of IFT was 10.5 cm (mean). The mean traction force was 16.28 kg. Total medialization was significantly higher during the first 15 min of vertical fascial traction (*p* < 0.05).

**Conclusions:**

IFT provides significant medialization for the anterior rectus sheath in the cadaveric model. The findings align with results from a retrospective case study. Therefore, we see IFT as a beneficial tool in abdominal wall surgery.

## Introduction

The treatment of primary and secondary ventral hernias of the midline is mainly standardized concerning smaller defects. It is generally accepted that due to disturbances of the extracellular matrix mesh-based reconstructions are needed to provide long-lasting prevention of recurrence [1, 2]. Furthermore, it has been shown that the anatomical reconstruction of the abdominal wall is associated with improved function and reduced complication rate in comparison with bridging techniques [3, 4]. Whereas smaller defects in patients with compliant abdominal walls can be treated endoscopically, laparoscopically and by open approaches with almost comparable results concerning the recurrence rate, the repair of major defects or defects in noncompliant abdominal walls needs more extended techniques to achieve adequate myofascial advancement to reconstruct the anatomy of the abdominal wall. More than 30 years ago the anterior component separation technique (ACS) was described [5]. It has evolved since then using endoscopic approaches for the external release or completely open approaches sparing the perforans vessels because the original procedure was associated with a high wound complication rate [6–9]. A further step was introduced to reduce the complication rate by way of posterior component separation, which is based on the release of the transversus abdominis muscle, and well-known as TAR [10]. Although there are promising results, a comprehensive review could not establish any advantage in terms of surgical site occurrences of the TAR over more sophisticated ACS techniques [11].

The new development of robotic TAR as the most recent technique shows also promising results [12], however, it is not available everywhere. Nevertheless, the robotic approach means minimally invasive access, but the preparation planes in the abdominal wall as well as the extent of the preparation remains unchanged. A further aspect deserving more attention concerns any damage of the neurovascular bundles at the semilunar line during TAR. Of course, in primary repairs these structures can be spared easily during TAR by experienced hands, but in recurrent cases with scaring of the retro rectus space, it is sometimes more difficult. Until now there has been no study looking for any denervation after TAR which could be diagnosed or excluded by simple computed tomography.

Taking these considerations into account, a novel approach for the treatment of large ventral hernias and loss of domain hernias (LOD) was first described by Eucker et al. in 2017. Using the Abdominal Wall Expanding System (AWEX), myofascial advancement was achieved intraoperatively by stretching the abdominal wall using a retractor system and clamps [13]. Nevertheless, the method lacks standardization and reproducibility specifically because no quantifiable traction is applicable. By using a newly developed device called fasciotens®Hernia, which provides quantifiable traction, the method has evolved and is now known as intraoperative fascial traction (IFT) [14]. In a few clinical series’ using standardized fascial traction with Fasciotens® the effectivity to reconstruct major midline defects could clearly be demonstrated [14, 15]. The myofascial advancement allows fascial closure in these studies in 90% of the cases (mean defect size 16.1 cm). Only in a few exceptionally large defects or further defects in the lateral compartment was an additional TAR necessary, leading to a low perioperative complication rate.

However, there is a lack of experimental data concerning the exact localization of the compartments where IFT really works and the effectivity of IFT depending on the localization of the defect. Furthermore, it is unknown how the traction forces change over time during IFT, especially in different phases of myofascial advancement.

To address this lack of experimental data and to obtain reproducible results, we used a cadaveric model with human specimens with intact abdominal wall. The present study addresses the following questions in particular:

1. How much myofascial advancement can be achieved by standardized IFT?
2. Which compartment of the abdominal wall provides myofascial advancement?
3. Time course of myofascial advancement.
4. Establishing a new method to exactly determine and measure the applied traction forces.

## Materials and methods

Four fresh frozen human specimens were obtained from the anatomical institute of the University of Hamburg. All included postmortem human specimens had consented to tissue donation for scientific purposes. Due to European and German regulations, no medical history of the human specimen was accessible. Despite a scar in loco typico of an open appendectomy in one human specimen, no other visible or palpable signs for previous abdominal surgery were found. One specimen was female, all others were male, with an age range between 71 and 92.

### Rives-Stoppa procedure

A midline skin incision was performed from the xyphoid to pubic bone. After subcutaneous dissection, the linea alba was identified and longitudinally incised. The rectus sheath was opened at the medial edge by a retro rectus dissection until the semilunaris line was identified.

### IFT

**Fig. 1:**
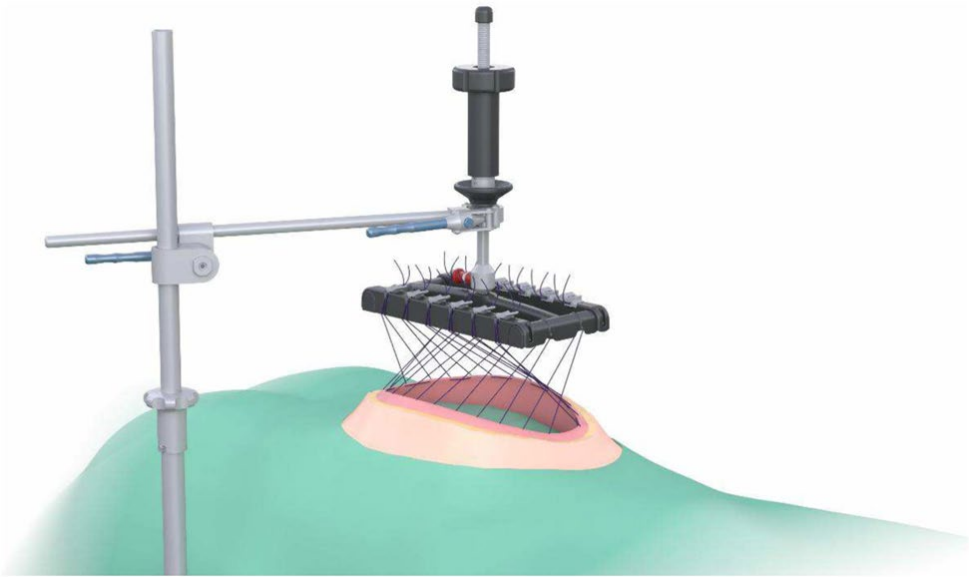
Assembled fasciotens^®^Carrier and fasciotens^®^Hernia

We proceeded by stitching six sutures (Vicryl^™^ USP 1, Ethicon) as U-sutures 1 cm lateral to the edge of the anterior sheet of the rectus sheath. The stitch length was 2 cm, and the U-sutures were equally distributed from cranial to caudal. The sutures were crossed over one another and after assembly of the traction device (fasciotens^®^Carrier and fasciotens^®^Hernia—see Fig. 1), they were attached to the suture retention frame. IFT was started with a traction force of 14 kg which was increased during the experiment. (Mean 16.28 kg). The fascial traction was carried out for a total of 30 min. Every two minutes the sutures were retightened as described by Niebuhr et al. to maintain the tension applied on the anterior rectus sheet [14, 15].

**Fig. 2:**
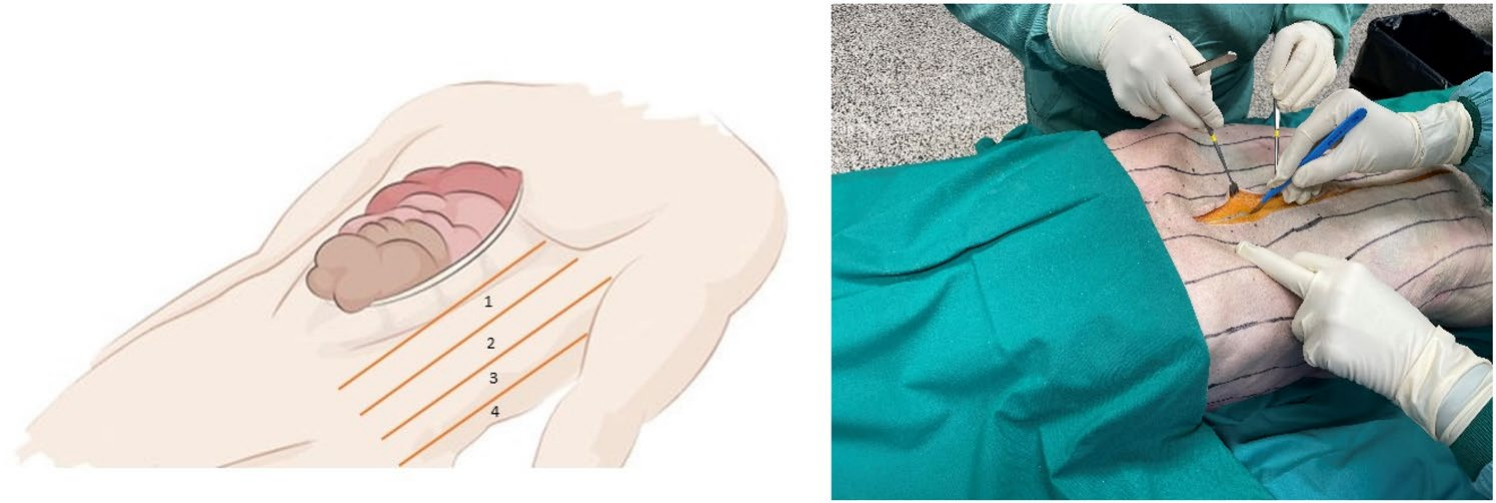
Measurement compartments

For measuring the medial advancement of the anterior rectus sheet, vertical lines were marked on the skin at 4 cm intervals beginning at the midline extending to the transverse process of the spine (see Fig. 2). Thus, the abdomen was separated into compartments. The compartments were numbered, starting most medially. Because a subcutaneous dissection of the skin and fat from the anterior sheet of the rectus muscle was not carried out, the myofascial advancement was measurable on the skin. After 15 and 30 min of vertical diagonal traction, the distance between the vertical lines was measured at 3 different marks parallel to the midline incision. The mean value of the three different marks was used for data collection. The medial advancement was determined using the delta at 15 and 30 min. Measurement was done separately for both sides.

**Fig. 3:**
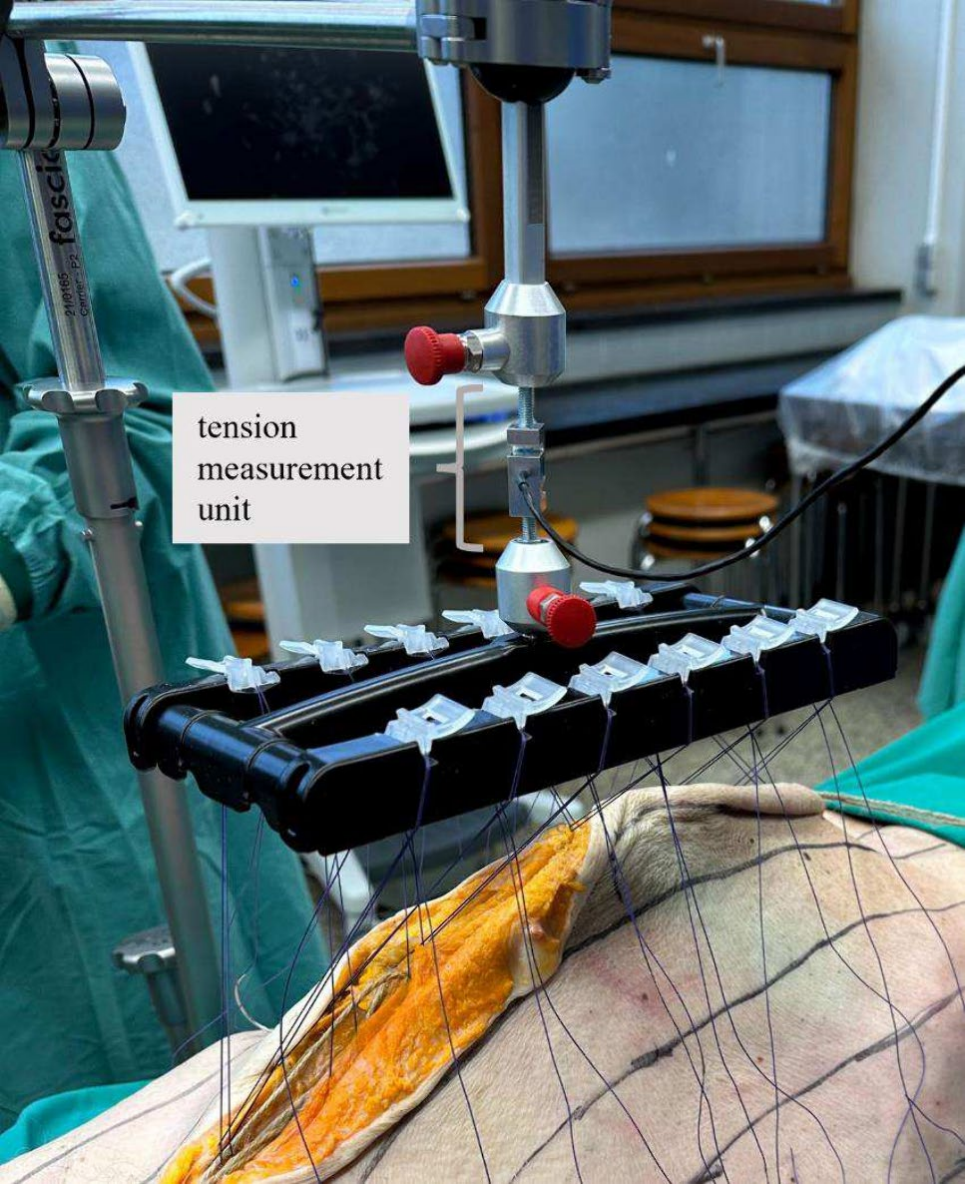
fasciotens^®^Hernia with tension measurement unit

Furthermore, the tension that acted on the fascia via the traction device (fasciotens^®^Hernia) was measured in one specimen as described below. A tension measurement unit (in-line load cell) was screwed in between the suture retention frame and the traction controller (fasciotens^®^Hernia) (see Fig. 3). The custom-made in-line load cell is an assembly of a load-cell KD24s (Me-Messsysteme, Hennigsdorf, Germany), an amplifier (GSV-11; Me-Messsysteme), a data acquisition device NI USB-6008 (National Instruments, Munich, Germany) and a custom-made data acquisition software programmed with Labview 19 (National Instruments). The tensiometer allows monitoring of the tension applied by the traction controller over time. The tension values were converted into kg as customary for the fasciotens^®^Hernia device.

## Results

### Total medialization

For all specimens, medial advancement was measured separately for both sides using the marked lines and compartments as described above. Total medialization after 30 min of IFT was 10.5 cm in the mean (5.5 cm for the left side and 5 cm for the right side). The mean values and standard deviations can be seen in Table 1. It was observed that the medial advancement differs between the first 15 min of IFT and the second 15 min. For the right side, the medialization during the first 15 min of IFT was significantly higher than between 15 and 30 min (*p* = 0.003; *α* = 0.05). The same pattern was seen for the total medialization including both sides (*p* = 0.03; *α* = 0.05) For the left side alone, no significant difference was observed (*p* = 0.65; *α* = 0.05).

**Table 1:** Mean values and standard deviations for medialization of the anterior rectus sheets

	Left [cm]	Right [cm]	Both sides [cm]
Myofascial advancement (Mean cumulative)	5.5	5	10.5
Myofascial advancement (Mean cumulative) after 15 min	3	4	7
Myofascial advancement (Mean cumulative) after 30 min	2.5	1	3.5
Myofascial advancement (Standard deviation cumulative)	2.291	1.225	3.354
Myofascial advancement (Standard deviation cumulative) after 15 min	1.414	1.225	2.550
Myofascial advancement (Standard deviation cumulative) after 30 min	1.118	0.000	1.118

**Fig. 4:**
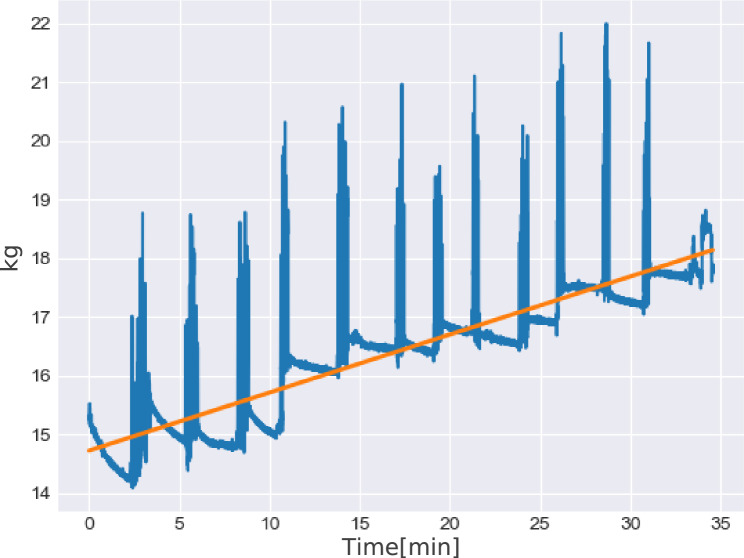
kg during traction. The orange line shows the regression line

When the most medial compartment (compartment 1) is compared to the most lateral compartment (compartment 4) a significant difference in the amount of myofascial advancement can be seen (mean values 2.375 vs 0.5; *p* = 0.0002; *α* = 0.05). To achieve optimal results using IFT, the sutures, which distribute the traction force from the suture retention frame to the fascia, must be kept in tension. Therefore, the sutures were retightened, as described by Niebuhr et al., every 2 min [14, 15]. Figure 4 shows the applied traction force during 30 min of IFT. The high peaks seen every 2 min are the retightening of the sutures. The orange line shows the regression line.

### Traction forces

**Fig. 5:**
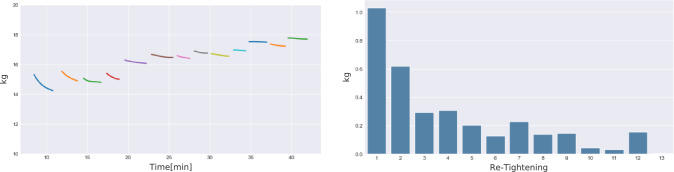
Traction force curves (regression) and delta-f for traction forces during every 2-min interval

Figure 5 shows the curve of traction force during each 2-min interval. Furthermore, the delta-f (change in traction force) for each 2-min interval are shown. Corresponding to the lower medial advancement between 15 and 30 min of IFT, the delta-f become smaller during IFT. Furthermore, the traction forces increase over time during the 30 min of IFT (Table 2).

**Table 2:** Traction forces during 2-min intervals and overall mean

Traction interval	1	2	3	4	5	6	7	8	9	10	11	12	13	overall
Traction force (mean) [kg]	14.61	15.14	14.86	15.14	16.15	16.53	16.47	16.79	16.62	16.93	17.50	17.27	17.72	**16.28**

## Discussion

Abdominal wall defects still represent major surgical challenges because of the necessity of extended surgical procedures with relevant postoperative complications. Recently, Eucker et al. published long term results of IFT in 33 patients with very promising results after a median observation time of 29 months [16]. It should be noted that the traction force used in Eucker’s AWEX-system is not quantified or standardized. Nevertheless, the median myofascial advancement was 12 cm without any form of component separation. Therefore, IFT has proven to be a helpful tool for lengthening the abdominal wall and increasing the intraabdominal volume. Furthermore, in most cases the indication for a component separation was no longer necessary. Niebuhr et al. showed similar effects (mean 10.2 cm) using the standardized fascial traction using the fasciotens^®^ system [15]. However, it must be noted that additional steps are required intraoperatively, including suturing the sutures to the anterior sheets of the rectus sheaths, assembling and attaching the device and 30 min of controlled fascial traction.

To provide experimental data concerning the effect of IFT, we applied the fasciotens^®^ system in a cadaveric model. The traction started at time point zero with 14.61 kg. With lengthening of the abdominal wall, the traction force decreased, which is shown in Fig. 5 and is easily seen by the loosening of the sutures. Every 2 min the sutures were retightened. As shown in Fig. 4 the traction force is lost very quickly at the beginning. Furthermore, the medialization of the abdominal wall was significantly higher in the first 15 min of IFT. We conclude that the lengthening of the abdominal wall is mostly pronounced at the beginning of traction. Table 1 demonstrates the mean myofascial advancement of 10.5 cm after 30 min. However, 7 cm are already gained after 15 min. In accordance with the myofascial advancement, the loss of tension decreased rapidly during the traction period of 30 min. Interestingly, the delta-f (change in traction force) are considerably higher in the beginning of IFT and, except for one outlier; strives towards zero after 30 min (see Fig. 5). In accordance with the higher gain in myofascial advancement during the first 15 min of IFT, we believe that this could be explained by the stretching tendency of the abdominal wall. The overall traction force increased to about 18 kg during the procedure. Although it would be expected that the oblique and transverse muscles (corresponding to marked compartments 3 and 4) may be more suitable for diagonal traction than the rectus muscle compartment (corresponding to marked compartments 1 and 2). Interestingly, the myofascial advancement was significantly higher in the medial compartment (corresponding to marked compartment 1) compared to the most lateral compartment (corresponding to marked compartment 4). Nevertheless, it is unclear whether these findings can be transferred to a pathological situs (e.g., LOD hernias) in vivo where the abdominal wall is retracted.

As to our knowledge there are only a few studies measuring the effect of component separation techniques on the lengthening of the abdominal wall in cadaveric models [17–19]. Majumder et al. investigated the mobilization of the anterior rectus sheath after anterior or posterior component separation [19]. The authors tried to also establish any difference of the effect at the upper, middle, and lower abdomen. Principally, the myofascial advancement was significantly more pronounced after the posterior release compared with the anterior approach, at least in the upper and middle section of the abdominal wall. The median gain of length of the anterior rectus sheath was 8.8 cm after anterior release and 10.2 cm after posterior release, respectively.

Sneiders et al. also compared the myofascial mobilization after anterior and posterior component separation techniques and demonstrated a slight superiority of the anterior release concerning the anterior rectus sheath, whereas the posterior release provided more mobilization of the posterior rectus sheath [17, 18]. The overall mobilization, however, was significantly lower than the one described by Majumder et al.

In contrast to these studies, we determined the lengthening of the whole abdominal wall as shown in the methods section. Nevertheless, the gain by IFT is comparable to the effect of a posterior release and more effective than an anterior release when compared with the results of Majumder et al. The results of Sneiders et al. seem to be very logical but are less effective compared to those obtained by IFT. The synopsis of these studies underlines that IFT leads to at least a comparable mobilization of the anterior rectus sheath with any form of component separation techniques at least in cadaveric models.

Therefore, IFT may be an alternative to component separation related to the goal of abdominal wall reconstruction. An argument favouring the posterior release concerns the possibility of using exceptionally large meshes. As to our knowledge there is no data whether we really need the large overlap after the posterior release except in the presence of any additional lateral defect. Since IFT also lengthens the medial compartment, i.e. the compartment of the rectus muscle, the size of a retro-rectus mesh after IFT is larger and should be sufficient to ensure adequate reinforcement of the posterior layer of the rectus sheath. Reducing the frequency of component separation techniques will represent a benefit for the patient in terms of a reduced complication rate by a smaller surgical trauma. Although there are some hints from clinical data concerning the reduced complication rate after IFT, a randomized study comparing IFT and TAR would be desirable, especially concerning the function of the abdominal wall even after a longer observation period. To clarify these questions, the University of Aachen has started a multicentre randomised controlled trial (RCT) with two patient groups in which IFT and TAR are compared. In addition, the authors have initiated an ongoing follow-up study focussing on the long-term results of IFT.

### Limitation of the study

First, it is not known whether the cadaveric abdominal wall is equal to the patient’s one in terms of elasticity and individual compliance. Furthermore, in a clinical setting any incomplete relaxation of a patient will strongly interfere with the effect of IFT. In addition, a standardised measurement of the tension of the abdominal wall and in particular the anterior rectus sheaths before and after IFT would be desirable. Until now any microscopic damage of the different layers of the abdominal wall after IFT has not been investigated as well as the function of the abdominal wall, especially in comparison with the component separation technique.

## Conclusion

In conclusion the questions initially addressed could be answered by this cadaveric model. The mean myofascial advancement reaches about 10 cm after IFT in a cadaveric model which is comparable with the clinical results achieved in patients with abdominal wall defects [13–15]. Furthermore, the medial and lateral compartments of the abdominal wall are accessible to IFT. However, in our cadaveric model the myofascial advancement was higher in the medial compartment. The most pronounced effect of abdominal wall lengthening is observed during the first 15 min, but relevant further mobilization can be achieved until 30 min. The procedure to measure the exact traction forces presented in the method section is appropriate in a cadaveric model. Additional studies to transmit the measurement procedure and the myofascial advancement in vivo are needed.

## Declarations

### Image rights

The animated image used in Fig. 1 belong to Fasciotens GmbH, Essen, Germany and have been used following appropriate authorization. The images used in Figs. 2 and 3 belong to Frederik Morgenroth, Hamm-Lippstadt University of Applied Sciences, Hamm, Germany.
